# Multilabel Image Annotation Based on Double-Layer PLSA Model

**DOI:** 10.1155/2014/494387

**Published:** 2014-06-04

**Authors:** Jing Zhang, Da Li, Weiwei Hu, Zhihua Chen, Yubo Yuan

**Affiliations:** ^1^School of Information Science and Engineering, East China University of Science and Technology, Shanghai 200237, China; ^2^State Key Lab. for Novel Software Technology, Nanjing University, Nanjing, China

## Abstract

Due to the semantic gap between visual features and semantic concepts, automatic image annotation has become a difficult issue in computer vision recently. We propose a new image multilabel annotation method based on double-layer probabilistic latent semantic analysis (PLSA) in this paper. The new double-layer PLSA model is constructed to bridge the low-level visual features and high-level semantic concepts of images for effective image understanding. The low-level features of images are represented as visual words by Bag-of-Words model; latent semantic topics are obtained by the first layer PLSA from two aspects of visual and texture, respectively. Furthermore, we adopt the second layer PLSA to fuse the visual and texture latent semantic topics and achieve a top-layer latent semantic topic. By the double-layer PLSA, the relationships between visual features and semantic concepts of images are established, and we can predict the labels of new images by their low-level features. Experimental results demonstrate that our automatic image annotation model based on double-layer PLSA can achieve promising performance for labeling and outperform previous methods on standard Corel dataset.

## 1. Introduction

With the advent of the information era, the usage of the Internet is increasingly prevalent and the scale of the multimedia database is fast growing. How to organize, index, and retrieve the image data set has become an important issue and absorbed more attention in recent years. The existence of the semantic gap [1] leads to the fact that the images with similar visual characteristics may not be similar in semantics. For solving this problem, many image automatic annotation methods have been proposed for large-scale image understanding. Inspired by the techniques of text analysis, probability topic models are used to learn the relationships between the low-level visual features and high-level semantic concepts for automatic image annotation. Using probability topic model can effectively map high-dimensional image feature vectors to a low-dimensional space, greatly reducing the redundant information of the image and the time complexity of the algorithm. The widely used topic models include Latent Dirichlet Allocation (LDA) [2] and Probabilistic Latent Semantic Analysis (PLSA) [3]. The LDA topic model exploits complex bayesian structure and needs to determine prior parameters of the model, which makes its applicability less wide than PLSA model.

## 2. Related Work

Automatic image annotation is mainly to predict the semantic labels according to the visual content of images, which can be roughly divided into two categories [4]. The first one regarded automatic image annotation as a supervised classification problem. Specifically each word is viewed as a unique class and a binary classifier is trained for each class, or a multiclass classifier is trained by low-level features independently to predict the labels of unlabeled images. The second one represents the labels and visual features of images as different semantic spaces, in which the correlations between visual content and text labels are trained by the labeled images. Then the semantic concepts of unlabeled images will be predicted via statistical inference.

The first category is developed into the machine translation and multimodel fusion method in recent years from exploiting the simple SVM (support vector machine) or GMM (Gaussian mixture model) for image classification in early years. In 2002, the translation model (TM) was proposed by Duygulu et al. [5], in which an image is cut into regions and each region corresponds to an object. Then these regions are clustered into blobs based on the features of regions, and labeling can be regarded as a process which translates blobs into labels. Kobus Barnard et al. [6] proposed a multimodel annotation algorithm which fused hierarchical clustering model, TM, and LDA model with information of different aspects. While the method considers annotation of both the whole image and the image regions, the joint probability distribution of blobs and keywords is obtained by learning, and the image annotation and regional annotation problems are translated into the associated problems among images, regions, blobs, and keywords.

The cross media relevance model (CMRM) proposed by Jeon et al. [7] also applies the segmentation regions to represent the image. Different from TM, this method considers that the keywords of the image do not get one-to-one correspondence to regions, and the images annotation is realized by learning the joint probability distribution between the keywords and regions of image. Lavrenko et al. [8] proposed continuous-space relevance model (CRM), which can be trained and modeled on the continuous features. And it is not dependent on the clustering of the low-level features, which makes it will not be affected by the clustering granularity and can achieve better performance. In the early-stage work, we also proposed an image annotation algorithm based on multiple models [9]. Two different models are used to analyze semantic concepts of foreground and background: Multiple Nystrom-Approximating Kernel Discriminant Analysis and Region Semantic Analysis, and then Latent Semantic Analysis assists in amending the annotation for error correction.

The most important part of the second category is to learn the link between the low-level visual features and high-level semantic features by the probability topic model. Blei and Jordan [10] model the keywords and image by correlation LDA model. Firstly, a series of latent topics are generated by associate visual features with semantic concepts, and image is decomposed into a series of collections of the latent topics. Then, a subset selected from these latent topics is converted to a number of hybrid models based on LDA, through which the image semantic annotation is generated. PLSA-WORDS annotation algorithm is proposed by Monay and Gatica-Perez [11], in which asymmetric learning algorithm is used to learn a latent space from text data and maintains the relation with visual features and achieves fairly good performance of image annotation and retrieval.

PLSA-FUSION [12] algorithm proposed by Li et al. learns not only the latent topics in the label aspect, but also the latent topics in the visual aspect. Then these two latent semantic topics are weight-fused through an adaptive asymmetric learning method, and a new model is generated, which can predict semantics of the unlabeled images. Akcay and Aksoy also adopt PLSA model [13] to detect the remote sensing image. They combined Principal Component Analysis (PCA) with mathematical morphology method for remote sensing image segmentation. Next they extracted features from the segmented regions and applied PLSA model from the perspective of pixels to measure the similarity between image regions. Then the segmented regions are clustered, and the information of spectrum and structure are combined to realize the semantic detection of remote sensing image. Different from above algorithms, Zhuang et al. analyzed PLSA model from a semisupervised perspective and applied it to image classification [14].

Traditional PLSA based image annotation models usually encounter a problem that the scale of visual and text words do not match [15]. Namely, the scale of vocabulary of text features is usually the power to 10, but that of vocabulary in visual aspect is up to about 500. So during analyzing, deviation will be produced. However, Semisupervised PLSA overcomes this problem well, which added label information of images into the EM algorithm for the calculation of model parameters.

We propose a new multilabel annotation method of images based on double-layer PLSA model, which applies relevant knowledge of latent topic space for image semantic annotation. In this method, we represent the image visual features by BoW model [16] and generate the latent semantic topic spaces through using first-layer PLSA, respectively, from the visual space and text space. Then two topic subspaces are integrated by second-layer PLSA, and the top layer latent semantic topics are obtained to realize the connection between the low-level visual features and high-level semantic concepts. Experimental results based on Corel5K illustrated that this method can effectively narrow down the semantic gap and achieve better performance on labeling multilabel image.

The rest of the paper is organized as follows.  Section 3 presents the double-layer PLSA model and the image automatic annotation algorithm is introduced in detail in Section 4. Experiments and results analysis are illustrated in Section 5, followed by conclusions in Section 6.

## 3. Image Annotation Model Based on Double-Layer PLSA

**Figure 1:**
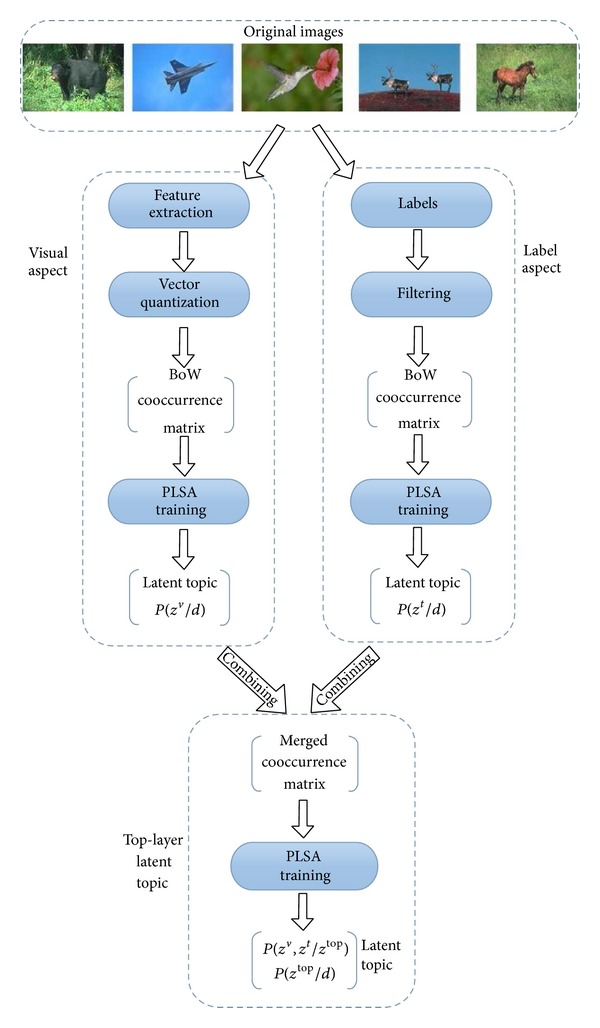
The flow diagram of the double-layer PLSA model.

Image automatic annotation algorithm based on double-layer PLSA fully utilizes the visual and semantic information of images to construct the annotation model by using PLSA. The second-layer latent semantic topics are looked on as the “bridge” to connect the visual features and semantic concepts. The framework of our annotation model is illustrated in Figure 1. There are two main parts in this framework, including representation of the image content and double-layer PLSA model. We will introduce them in detail as follows.

### 3.1. Representation of the Image Content

The model of BoW is widely applied in the natural language processing and information retrieval, in which texts (sentences or documents) are taken as unordered data sets and the influence of the word order is ignored. Nowadays this model is widely used in the field of computer vision. In our annotation model, BoW model is also used to represent the image content and the processing procedure is described as follows.

In the semantic label space, the limited vocabularies annotated manually are looked upon as a label set, which is originally a series of unordered keywords set and can be represented by BoW directly. Suppose that the number of the given label *t* is *N*_*t*_; then the label information of the image *I*_*i*_ can be represented as a vector with *N*_*t*_ dimensions, illustrated as follows:

(1) $$\begin{array}{c} T\left(I_{i}\right)=\left\{n\left(I_{i},t_{1}\right),\ldots ,n\left(I_{i},t_{j}\right),\ldots ,n\left(I_{i},t_{N_{t}}\right)\right\}, \end{array}$$

where, *n*(*I*_*i*_, *t*_*j*_) is the quantity of the label *t*_*j*_ appearing in the given image *I*_*i*_; usually the values are 0 or 1.

In the visual feature space, the image is divided into small pieces with same size, and visual features of the small pieces are extracted and clustered to generate a visual vocabulary. Then image can be simply expressed as a collection of several visual words. Considering that the different low-level features of images can express various aspects of image content, and each has its advantages in specific aspects, so the combination of different features is a wise method to minimize the loss of feature discretization. Assuming that the *N*_*v*_ is the amount of category, which is gained by clustering, each cluster can be represented by a visual word. All visual words make up the vocabulary, and an image *I*_*i*_ can be represented as a visual word histogram, which can be expressed as a vector with *N*_*v*_ dimensions and illustrated as (2). Consider

(2) $$\begin{array}{c} V\left(I_{i}\right)=n\left(I_{i},v_{1}\right),\ldots ,n\left(I_{i},v_{i}\right),\ldots ,n\left(I_{i},v_{N_{v}}\right), \end{array}$$

where *n*(*I*_*i*_, *v*_*i*_) represents the number of visual word *v*_*i*_ in the given image *I*_*i*_. Unlike the elements of the vector in (1), *n*(*I*_*i*_, *v*_*i*_) can be any integers greater than 0, and the combination of different visual features can be simply connected via feature vectors [17].

**Figure 2:**
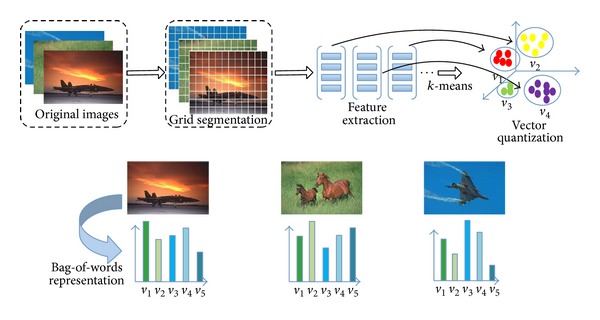
The flow chart of getting BoW representation of the image low-level feature.

The BoW model provides an unified form to represent the image content, in which each feature plays an effective role in representation of image content.  Figure 2 represents how to get the BoW representation of image low-level visual features. The first part describes grid segmentation, feature extraction, and the feature vectors quantization. The second part illustrates the process of image representation by BoW model.

To construct BoW representation by visual features, three steps are implemented as follows. Firstly, we extract the pyramid gradient direction histogram feature (Pyramid Histogram of Oriented Gradients, PHOG) [18, 19] on the whole image and obtain PHOG histogram. Secondly, the image is segmented into fixed grids, and the scale color descriptor (SCD) and Gabor texture feature are extracted for each small grid. Then, *k*-means is used to cluster the SCD and Gabor features of all grids, respectively, and the visual words are achieved. Finally, these two categories of visual word vectors are fused, and the BoW representation of each image is gained.

**Figure 3:**
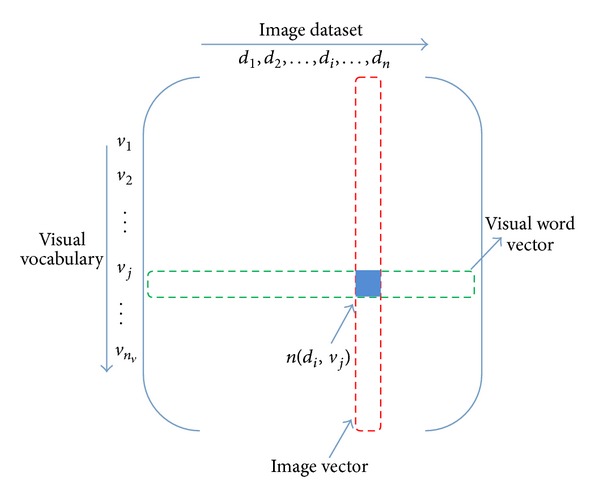
The “image visual-word” matrix of the image low-level feature.

Through the BoW model the continuous low-level feature information of image is transformed into the discrete form, so that each image can be represented by the visual vocabulary vector simply. By putting all the feature vectors together, we can get the visual word cooccurrence matrix of the whole image set and the specific form is shown in Figure 3. Each column of the matrix represents an image vector, in which *n*(*d*_*i*_, *v*_*j*_) represents the number that occurrence of visual word *v*_*j*_ appeared in image *d*_*i*_.

### 3.2. Model of Double-Layer PLSA

According to the framework of double-layer PLSA model illustrated in Figure 1, the low-level features and labels in an image can be transformed to two different cooccurrence matrixes; then PLSA model is used to analyze the visual information and semantic information, respectively. Therefore, the low-level feature space and the high-level semantic space are mapped to two latent topic subspaces. However, there is no link between the two topic subspaces.

In order to achieve the labels of image semantics, the most important step is to establish the connection between the high-level semantics and the low-level features.

Assuming that the image set is *D* = {*d*_1_, *d*_2_,…, *d*_*i*_,…, *d*_*n*_}, and the vocabulary (visual-words or labels) set is *W* = {*w*_1_, *w*_2_,…, *w*_*j*_,…, *w*_*m*_}. then *N* = {*n*(*d*_*i*_, *w*_*j*_)} represents the cooccurrence matrix of image visual-word and *n*(*d*_*i*_, *w*_*j*_) is the frequency of the visual word *w*_*j*_ in the image *d*_*i*_. In the analysis process of image by PLSA, a latent topic space *Z* = {*z*_1_, *z*_2_,…, *z*_*k*_} is introduced to map the high-dimensional image visual-word cooccurrence matrix to a low-dimensional latent semantic topic space. At the same time the abstract relationship would be explored, and the conditional probability of the “image visual-word” can be described as follows:

(3) $$\begin{array}{c} P\left(d_{i},z_{k},w_{j}\right)=P\left(d_{i}\right)P\left(\frac{z_{k}}{d_{i}}\right)P\left(\frac{w_{j}}{z_{k}}\right). \end{array}$$

**Figure 4:**
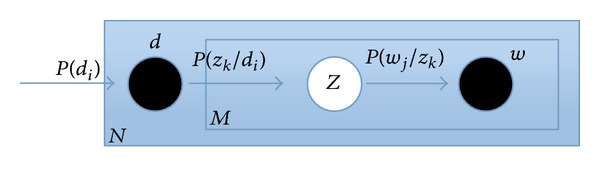
The graph representation of PLSA model.

The joint probability of a word (or visual word) *w*_*j*_ with image *d*_*i*_ is a marginalization over the topics *z*_*k*_ as

(4) $$\begin{array}{c} P\left(d_{i},w_{j}\right)=P\left(d_{i}\right)\overset{K}{\underset{k=1}{\sum }}P\left(\frac{z_{k}}{d_{i}}\right)P\left(\frac{w_{j}}{z_{k}}\right), \end{array}$$

where *P*(*z*_*k*_/*d*_*i*_) is the conditional probability of the latent topic *z*_*k*_ given the image *d*_*i*_, and *P*(*w*_*j*_/*z*_*k*_) represents the conditional probability of the visual word *w*_*j*_ given the latent topic *z*_*k*_. Moreover, PLSA can be illustrated in Figure 4; the nodes in the rectangular box express the three key elements “image” *d*, “latent topic” *z*, and “label” *w*, where the black nodes represent the observable random variables, and the white node represents the unobservable random variable.

**Figure 5:**
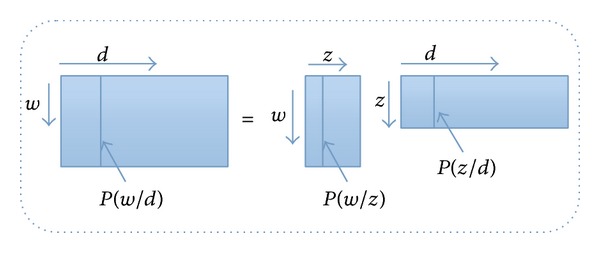
The matrix decomposition of PLSA model.

If we regard the cooccurrence matrix of “image visual-word” as a *N*∗*M* matrix, then the matrix decomposition of PLSA can be illustrated in Figure 5.

The most direct way of that apply PLSA model to the image understanding is combining the “visual-word image” matrix *N*_*M*∗*N*_*v*__ and “label image” matrix *N*_*M*∗*N*_*t*__ into a new matrix *N*_*M*∗(*N*_*v*_+*N*_*t*_)_, in which “visual-word image” matrix and “label image” matrix has the same format. But in this way it will arise a new problem of that scale is not consistent. In general, each image usually has thousands of visual words, but in most cases the labels of the image will not be more than 20. Therefore, the visual vocabulary is in a dominant position, which affects the results much more.

Although some normalization methods can alleviate the adverse effects, the normalization process needs a lot of experiments to determine the proper weight of different topics, which increases the time complexity of the algorithm. Therefore, in this paper we bring forward a double-layer PLSA model to solve this problem, and the specific process of the model is shown in Figure 1.

In the double-layer PLSA model the first layer includes two PLSA models, which conduct the latent semantic analysis, respectively, on image visual feature space and label space. The further analysis on latent semantic topics obtained from the first layer PLSA are processed to extract the second-layer latent semantic topics for forming the connection between low-level visual features and high-level semantic concepts.

**Figure 6:**
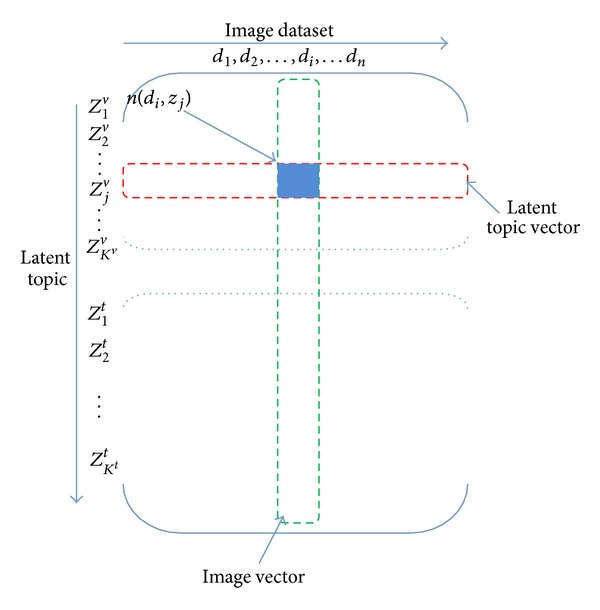
The cooccurrence matrix of image latent-topic.

As shown in Figure 1, the input of the second layer PLSA is cooccurrence matrix merged by the two latent topic matrixes generated from the first layer PLSA. As shown in Figure 6, in this merged cooccurrence matrix the column vectors are still the image collections *D* = {*d*_1_,…, *d*_*n*_}, and the row vectors are the two latent topic subspaces *Z* = {*z*_1_,…, *z*_*i*_,…, *z*_*K*_} (*Z* = {*z*_1_^*v*^,…, *z*_*K*^*v*^_^*v*^, *z*_1_^*t*^,…, *z*_*K*^*t*^_^*t*^}) obtained from the first layer PLSA. *z* ^*v*^ represents the latent topic distribution of the visual features, and *z* ^*t*^ represents the latent topic distribution of the labels. *n*(*d*_*i*_, *z*_*j*_) represents the probability of the first layer latent topic *z*_*j*_ in given document *d*_*i*_. It is obvious that the image latent-topic cooccurrence matrix has the same form as the previous image visual-word cooccurrence matrix (as Figure 3); hence the PLSA model can be used again.

**Figure 7:**
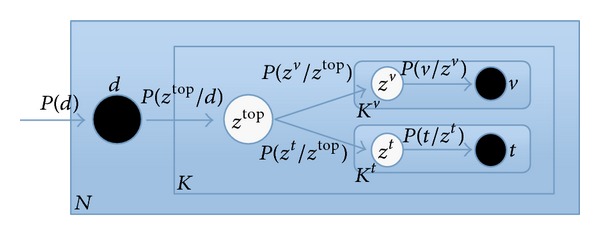
The calculating principle of the double-layer PLSA.

The calculation principle of the double-layer PLSA model is shown in Figure 7. There are 6 nodes in the rectangular box, including black nodes which represent observable random variables: images *d*, visual words *v*, and text labels *t*. White nodes represent unobservable random variables, namely, the latent topics, containing high latent topics *Z* ^top^, visual feature space latent topics *Z* ^*v*^, and the latent topics *Z* ^*t*^ of the labels. Figure 7 describes the corresponding relations between training parameters. Firstly select image *d* according to the probability *P*(*d*). Secondly obtain top layer topic probability distribution in the image *d* according to *P*(*z* ^top^/*d*); then get the label latent topic distribution of image *d* in the first layer according to *P*(*z* ^*t*^/*z* ^top^). Finally, get the text label distribution of image *d* according to *P*(*t*/*z* ^*t*^). The same to the visual aspect.

## 4. Image Annotation Algorithm

**Figure 8:**
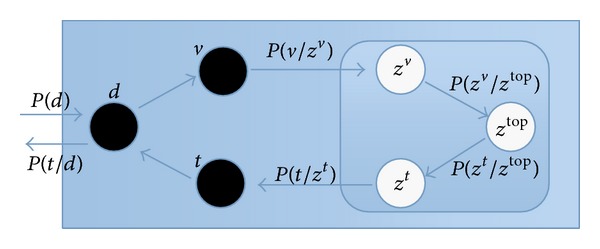
The tagging process of the image.

We analyze images from two aspects (*x*, *x* ∈ {*v*, *t*}, *v* represents visual feature, and *t* represents label) by double layer PLSA model. The image annotation algorithm and image annotation process are, respectively, shown in Algorithm 1 and Figure 8.

In terms of the related theory of PLSA and training process of double PLSA model, *P*(*v*/*z* ^*v*^), *P*(*t*/*z* ^*t*^), *P*(*v*/*z* ^top^), and *P*(*t*/*z* ^top^) are not restricted to specific image and can be applied to all other images. The folding-in [12] algorithm, as the simplified EM algorithm, is adopted to realize the auto-image-annotation. In this algorithm, the known parameters are kept unchanged in the iteration process, constantly updating unknown parameters until the maximum likelihood function gets the max value.

## 5. Experiments

In order to validate the effectiveness of image semantic content analysis by the proposed model, experiments have been done on Corel5K, and the experimental results are compared with other algorithms on the same image set.

### 5.1. Image Database and Evaluation Measures

Corel5K is currently the widely used image set in image retrieval and annotation field, which contains 5000 images with 50 different categories, and each category has 100 copies with the similar high-level image semantics. The images in Corel5K have a total of 371 labels, which are defined by LSCOM. Most of these labels occurred frequently in image set, but there are several labels, such as “pool,” “farms,” and “coast,” and that only occurred in 7 images. In order to reduce the influences of low-frequency words, we removed the tags appearing less than 8 times and finally constructed the ideal vocabulary with 260 labels [20].

We compared the annotation results of our double-layer PLSA model with the ground truth to verify the effectiveness of the proposed algorithm. The evaluation method based on label is used, including “recall” and “precision” of each label. In this experiment, we only took labels with the top five largest posterior probabilities as annotation result of each image and calculated the precision and recall for each label. For a given label *w*, the calculating formula of precision and recall are shown in the following:

(5) Precision=NWTNW,Recall=NWTNWGT,

where *N*_*WT*_ is the correct number of *w* annotated by this algorithm, *N*_*W*_ represents the number of images containing *w* after image annotation, and *N*_*WGT*_ is the number of image including *w* in the ground truth.

### 5.2. The Contents of Experiment

In our experiments the whole data set is divided into two parts of which 4500 images are taken as the training set and the rest of them as the testing set. The visual features of image are represented by BoW model. First, segment the image into small grids. If the scale of segmentation is small enough, all the content in the image can be expressed in detail. But at the same time, it may increase the computing complexity of the algorithm. If the scale is too large, the image content will not be represented accurately enough. According to our research results on the image content representation with different segmentation scales [21], we adopt 15∗15 fixed-size, which makes the image content representation accurate and the computing complexity of the algorithm ideal. In our experiments, we chose PHOG descriptor (Pyramid Histogram of Oriented Gradients, PHOG) [18, 19], SCD, and Gabor texture as low level features, in which PHOG histogram has 425 dimensions. By amounts of experiences, we found that if the cluster number of SCD and Gabor texture are 325 and 250, respectively, the dimensions of BoW model are 1000, which is the best way to represent the image content.

An important parameter of PLSA model is the number of the latent topics, which determines the time needed for model training to a large extent. If the predefined latent topics are too few, they may not be good enough to express the potential relationship between the visual information and concepts. However, if they are too many, it will take a lot of time for training and the efficiency of the model will decrease. Meanwhile, it may increase the possibility of over-fitting. Considering the amounts' difference between the text label sets and visual vocabulary, and the corresponding parameters value mentioned in the reference [12], we defined the number of latent topics of the label text as 120 and the number of latent topics of the visual as 80. Then we obtained a total of 200 latent topics after the first-layer PLSA analysis. On the second-layer PLSA processing, we found that the results with using 50 top-layer latent topics to learn “image latent-topic” co-occurrence matrix obtained in the first layer were best by large amounts experiments.

### 5.3. Experimental Results and Analysis

The experimental results on Corel5K by the proposed algorithm are shown in Table 1. For the 49 labels with the optimum performance, the double-layer PLSA achieved the satisfactory experimental results, in which the average recall and precision were both over 70%. But in the 260 high frequency label set for the reason of the imbalance of label distribution, the average recall and precision were reduced to 25% and 20%.

In order to express the advantage of double PLSA model, we compared our algorithm with the algorithm PLSA-FUSION proposed by Li et al. [12], TM [5], CMRM [7], CRM [8], and PLSA-WORDS [11] mentioned in paper [12]. In these experiments we used the same experiments data and evaluation method, and evaluated the results in two same label vocabularies: the 49 labels with the optimum performance and the 260 high frequency label sets.

**Figure 9:**
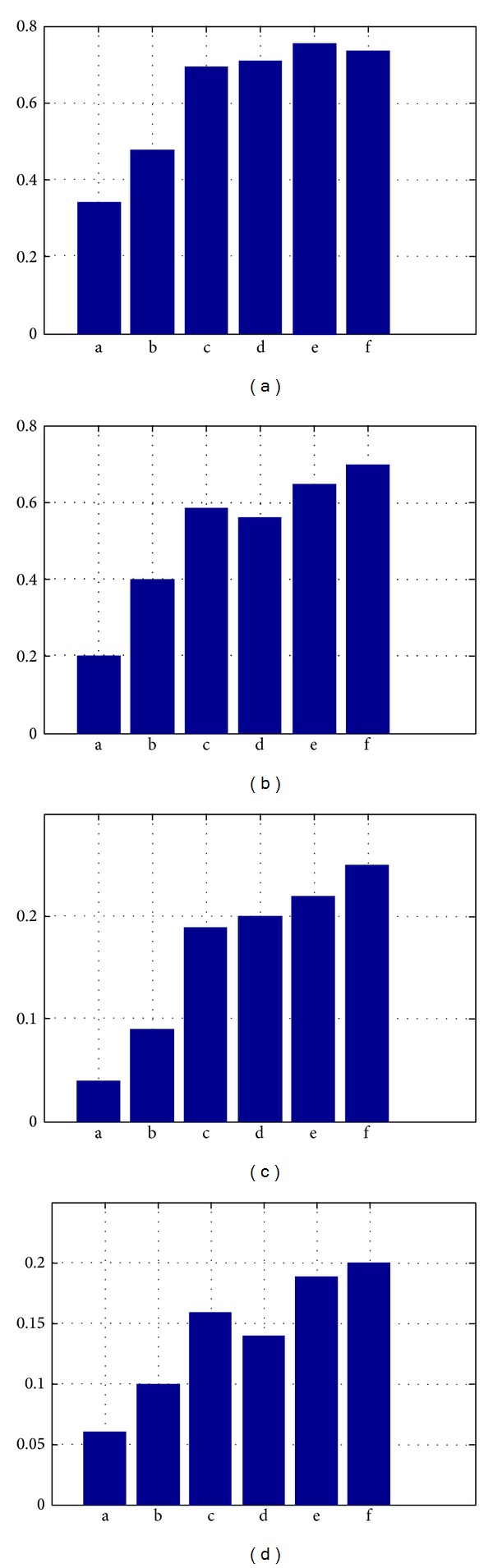
The comparison histogram of experiments ((a) the average recall of 49 labels, (b) the average precision of 49 labels, (c) the average recall of 260 labels, and (d) the average precision of 260 labels, where, a.TM b.CMRM c.CRM d.PLSA-WORDS e.PLSA-FUSION f.OURS).

Table 2 and Figure 9 illustrate the evaluation results of the proposed algorithm, TM, CMRM, CRM, PLSA-WORDS and PLSA-FUSION algorithms on the two predefined labels. It can be seen from the contrast histograms, the double-layer PLSA model obtained the promising performance on both two label sets. In the label set with 49 optimum labels, the average precision of the double-layer PLSA model exceeds all other algorithms, and the average recall is 2% less than that of PLSA-FUSION. In the set of 260 labels, the double-layer PLSA model outperforms all the other algorithms, which exceeded the PLSA-FUSION 3% and 1% on the average recall and precision, respectively.

## 6. Conclusion

In this paper, we analyzed the image content from the perspective of text and proposed an image multilabel annotation model based on a double-layer PLSA model. The low-level features of images are represented by BoW model, which converted continuous visual information into discrete visual histograms to represent the visual content of the image. Then the first-layer PLSA was used, respectively, in the label aspect and visual aspect to get two kinds of latent semantic topics. In the second-layer, PLSA was applied on these two unrelated latent semantic topic spaces to get the top-layer latent topics, which can create the connection between the visual features and labels. Finally, with the double-layer PLSA model, the image annotation was completed effectively. In order to prove the effectiveness of the double-layer PLSA model in image annotation, we completed experiments on Corel5K and compared with other related algorithms. The experimental results illustrate that the double-layer PLSA model can achieve outstanding performance for multilabel automatic annotation and outperform other related algorithms.

## Figures and Tables

**Algorithm 1:**
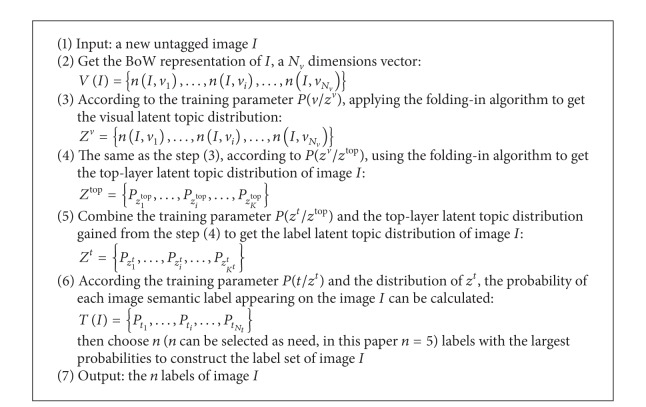
The image concept detection algorithm.

**Table 1:** Results on Corel5K by our algorithm: AR (average recall) and AP (average precision).

	49 labels		260 labels	
	AR	AP	AR	AP
Double-layer PLSA	0.74	0.70	0.25	0.20

**Table 2:** The results of contrast experiments: AR (average recall) and AP (average precision).

	49 labels		260 labels	
	AR	AP	AR	AP
TM	0.34	0.20	0.04	0.06
CMRM	0.48	0.40	0.09	0.10
CRM	0.70	0.59	0.19	0.16
PLSA-WORDS	0.71	0.56	0.20	0.14
PLSA-FUSION	0.76	0.65	0.22	0.19
Double-layer PLSA	0.74	0.70	0.25	0.20

## References

[1] Smeulders AWM, Worring M, Santini S, Gupta A, Jain R (2000). Content-based image retrieval at the end of the early. *IEEE Transactions on Pattern Analysis and Machine Intelligence*.

[2] Blei DM, David M, Andrew Y, Michael I (2003). Latent dirichlet allocation. *Journal of Machine Learning Research*.

[3] Hofmann T (2001). Unsupervised learning by probabilistic latent semantic analysis. *Machine Learning*.

[4] Wang Y, Mei T, Gong S, Hua XS (2009). Combining global, regional and contextual features for automatic image annotation. *Pattern Recognition*.

[5] Duygulu P, Barnard K, de Freitas N, Forsyth D, Heyden A, Sparr G, Nielsen M, Johansen P (2002). Object recognition as machine translation: learning a lexicon for a fixed image vocabulary. *Computer Vision*.

[6] Kobus Barnard K, Duygulu P, de Freitas N, Forsyth D, Blei D, Jordan MI (2003). Matching words and pictures. *Journal of Machine Learning Research*.

[7] Jeon J, Lavrenko V, Manmatha R Automatic image annotation and re-trieval using cross-media relevance modelsIn.

[8] Lavrenko V, Manmatha R, Jeon JA, Thrun S, Saul LK, Schölkopf B (2004). A model for learning the semantics of pictures. *Advances Neural Information Processing Systems*.

[9] Zhang J, Hu W Multi-label image annotation based on multi-model.

[10] Blei DM, Jordan MI Modeling annotated data.

[11] Monay F, Gatica-Perez D (2007). Modeling semantic aspects for cross-media image indexing. *IEEE Transactions on Pattern Analysis and Machine Intelligence*.

[12] Li ZX, Shi ZP, Li ZQ, Shi ZZ (2011). Automatic image annotation by fusing semantic topics. *Journal of Software*.

[13] Akcay HG, Aksoy S Automated detection of objects using multiple hierarchical segmentations.

[14] Zhuang L, She L, Jiang Y, Tang K, Yu N Image classification via semi-supervised pLSA.

[15] Hofmann T Probabilistic latent semantic indexing.

[16] Larlus D, Verbeek J, Jurie F Category level object segmentation by combining bag-of-words models and markov random fields.

[17] Wu L, Hoi SCH, Yu N Semantics-preserving bag-of-words models for efficient image annotation.

[18] Dalal N, Triggs W Histograms of oriented gradients for human detection.

[19] Lazebnik S, Schmid C, Ponce J Beyond bags of features: spatial pyramid matching for recognizing natural scene categories.

[20] Fang SL, Manmatha R, Lavrenko V Multiple bernoulli relevance models for image and video annotation.

[21] Zhang J, Zhao Y, Li D, Chen Z, Yuan Y Representation of image content of image content with multi-scale segmentation.

